# Association between trans-palmitoleic acid and metabolic dysfunction-associated steatotic liver disease-related hepatocellular carcinoma: NHANES 1999–2018

**DOI:** 10.3389/fnut.2025.1606482

**Published:** 2025-06-19

**Authors:** Qirui Li, Luqi Zhan, Qian Li, Shuai Zhu, Shuaihui Liu, He Jiang, Jinlin Cheng, Lanjuan Li

**Affiliations:** State Key Laboratory for Diagnosis and Treatment of Infectious Diseases, National Clinical Research Center for Infectious Diseases, National Medical Center for Infectious Diseases, Collaborative Innovation Center for Diagnosis and Treatment of Infectious Diseases, The First Affiliated Hospital, Zhejiang University School of Medicine, Hangzhou, China

**Keywords:** MASLD, HCC, trans-palmitoleic acid, NHANES, MASLD-HCC

## Abstract

**Introduction:**

Metabolic dysfunction-associated steatotic liver disease (MASLD) is a global health burden with an increasing incidence of hepatocellular carcinoma (HCC), yet early risk predictors remain elusive. This study investigated trans-palmitoleic acid (TPA) as a potential biomarker for MASLD-HCC risk.

**Methods:**

Using National Health and Nutrition Examination Survey (NHANES) data (*n* = 548), propensity score matching (PSM) minimized sociobehavioral confounders. Multivariable logistic regression and restricted cubic spline (RCS) models were employed to assess the association between TPA and HCC risk.

**Results:**

A striking nonlinear association between TPA and HCC risk was observed (*P*-nonlinear <0.001). Each unit increase in TPA elevated HCC risk by 52.4% (OR = 1.524, 95% CI = 1.397–1.677), with quartile analysis showing exponential risk escalation (Q4 OR = 753.7). Subgroup analyses identified heightened susceptibility in women (Q4 OR = 1148.83) and younger individuals (OR = 612.11). TPA correlated positively with lipid factors (triglycerides β = 1.236, LDL β = 0.557) and hematologic indices, while exhibiting a negative association with BMI (β = −0.037). Mediation analysis implicated triglycerides as a key metabolic intermediary (39.18% effect proportion).

**Discussion:**

These findings establish TPA as an independent MASLD-HCC risk factor with distinct demographic variability, potentially mediated through lipid dysregulation. While limited by observational design, this study highlights TPA’s prognostic value for HCC risk stratification, especially in non-diabetic populations (OR = 257.14). Future mechanistic studies should validate TPA’s oncogenic pathways and explore therapeutic targeting of trans-fatty acid metabolism to mitigate MASLD progression. The robust dose-response relationship and metabolic mediation effects position TPA as a promising candidate for incorporation into existing HCC prediction models.

## 1 Introduction

Metabolic dysfunction-associated steatotic liver disease (MASLD) has emerged as a global health crisis, affecting over 30% of the adult population worldwide (1–3). This condition represents a spectrum of liver disorders ranging from simple steatosis to progressive inflammation and fibrosis, with hepatocellular carcinoma (HCC) being the most devastating endpoint (4). The annual incidence of MASLD-related HCC has shown a concerning upward trajectory, contributing significantly to liver-related mortality and imposing substantial economic burdens on healthcare systems (5). Current epidemiological data reveal that MASLD-associated HCC accounts for 10%–30% of all HCC cases in Western countries (6–8), with projections suggesting this proportion will continue to rise in parallel with the obesity pandemic (9–11). The transition from MASLD to HCC involves complex pathophysiological processes that remain incompletely understood (12), highlighting the urgent need for mechanistic insights and predictive biomarkers.

Current diagnostic and therapeutic approaches for MASLD-HCC face significant limitations (13). Non-invasive diagnostic tools, including ultrasound and transient elastography, often fail to detect early malignant transformation (14, 15), while serum biomarkers such as alpha fetoprotein lack sufficient specificity for clinical implementation (16). Therapeutic strategies primarily focus on lifestyle modifications and metabolic control, with limited efficacy in preventing HCC development (17–20). Pharmacological interventions targeting metabolic pathways show promise but have not demonstrated consistent benefits in HCC prevention (4). The absence of reliable early warning indicators and incomplete understanding of the molecular mechanisms driving MASLD progression to HCC represent critical knowledge gaps in clinical hepatology. This underscores the necessity for identifying novel risk factors and elucidating their pathogenic roles in hepatocarcinogenesis.

A significant research gap exists regarding the role of trans-palmitoleic acid (trans-9-hexadecenoic acid, trans-C16:1 n-7, TPA), an understudied fatty acid metabolite, in MASLD-HCC development. This unusual fatty acid, primarily derived from dairy products, exhibits unique biological properties that distinguish it from other fatty acids (21). Interestingly, TPA levels have been associated with both beneficial and detrimental metabolic effects in epidemiological studies, creating a paradoxical scenario that warrants further investigation. While some reports link TPA to improved glucose metabolism (22), others highlighted a neutral role of TPA on diabetes (23), and others report elevated erythrocyte membrane TPA levels correlate with increased diabetes risk (24). Thus, understanding whether TPA serves as a biomarker of risk or an active participant in hepatocarcinogenesis could provide valuable insights for both risk stratification and therapeutic development.

To address these limitations, we employed a rigorous analytical approach utilizing data from the National Health and Nutrition Examination Survey (NHANES), a nationally representative dataset with extensive biochemical and clinical measurements. Our study design incorporated multiple statistical techniques: propensity score matching (PSM) to balance potential confounders across exposure groups (25); linear and nonlinear models [box plots, linear regression, and restricted cubic splines (RCSs)] to elucidate the dose-response relationship between TPA and HCC (26); subgroup stratification by gender, age and socioeconomic status to assess population differences in risk effects; and mediation effect models to dissect the mediating roles of various metabolic factors in the pathway linking TPA to HCC development (27). Our primary objectives were to establish the strength and nature of the association between TPA and MASLD-HCC, characterize population-specific risk patterns, and identify key metabolic mediators in this relationship. These findings may contribute to the development of novel risk prediction tools and inform targeted prevention strategies for high-risk populations.

## 2 Materials and methods

### 2.1 Data and preprocessing

The NHANES represents an ongoing, cross-sectional, and nationally representative investigation conducted in the United States. This significant research initiative is managed by the National Center for Health Statistics (NCHS) and receives authorization and funding from the Centers for Disease Control and Prevention (CDC) with the aim of evaluating the health and nutritional conditions of the civilian population in the United States who are not institutionalized. Data collection occurs biennially through a sophisticated multistage probability sampling methodology, which encompasses in-home, face-to-face interviews followed by comprehensive physical assessments at the Mobile Examination Center (MEC), during which blood and urine specimens are obtained. The NHANES protocol undergoes scrutiny and receives endorsement from the NCHS Research Ethics Review Board, ensuring that informed consent has been acquired from all participants involved. In our study, we utilized data from 10 cycles (1999–2018) of the NHANES database, which contains comprehensive test data on MASLD-HCC for participants. To account for inter-cycle variability, we applied survey weights provided in the NHANES documentation. Missing data were handled using multiple imputation methods. TPA level in plasma were measured by GC/MS as described in NHANES trans fatty acids procedure. The specific procedures included: converting fatty acids into their free forms for extraction, derivatization with pentafluorobenzyl bromide, separation via capillary gas chromatography, and detection by negative chemical ionization mass spectrometry. Fatty acids were identified by comparing retention times with standards and specific mass-to-charge ratios, while quantitative analysis was performed using a stable isotope-labeled internal standard method.

### 2.2 Diagnostic criteria for MASLD

The initial stage of MASLD is considered to be characterized by abnormal hepatic fat accumulation. Due to the absence of transient elastography data for liver assessment in the 1998–2018 NHANES database cycle, this study employs the Fatty Liver Index (FLI) as a surrogate assessment indicator. The FLI is calculated using the formula:


$$F\,L\,I=\frac{\begin{array}{c} e^{\left[0.953\times l\,n\,\left(T\,G\right)+0.139\times B\,M\,I+0.718\times l\,n\,\left(G\,G\,T\right)+0.053\times W\,C-15.745\right]} \end{array}}{\begin{array}{c} \left(1+e^{\left[0.953\times l\,n\,\left(T\,G\right)+0.139\times B\,M\,I+0.718\times l\,n\,\left(G\,G\,T\right)+0.053\times W\,C-15.745\right]}\right) \end{array}}\times 100$$

TG denotes total triglycerides, BMI refers to body mass index, GGT represents gamma-glutamyl transferase, and WC stands for waist circumference (28–32). After excluding other liver diseases associated with the aforementioned etiological factors, participants with a FLI > 60 were diagnosed with MASLD.

### 2.3 Patients inclusion criteria

**FIGURE 1:**
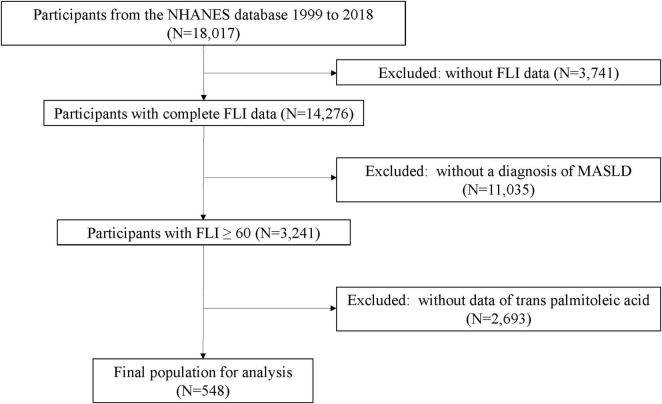
Flow chart of participant selection.

From the initial cohort of 18,017 participants, exclusions were made for the following reasons: (1) removal of samples lacking FLI data (*N* = 3,741), (2) exclusion of samples without MASLD (*N* = 11,035), and (3) exclusion of samples lacking TPA (*N* = 2,693). The final study population comprised 548 participants, including 239 cases of MASLD-HCC and 309 MASLD samples (Figure 1).

### 2.4 Propensity score matching

Propensity score matching is a statistical technique utilized to address data from observational studies. In observational studies, data bias and the presence of confounding variables are common due to various reasons. The method of PSM is designed to reduce the impact of these biases and confounding factors, thereby enabling a more rational comparison between the experimental group and the control group. This method was first introduced by Rosenbaum and Rubin (33) and is frequently employed in fields such as medicine, public health, and economics to match baseline characteristics (34). In our study, we implemented a 1:1 nearest-neighbor matching algorithm to ensure balanced group comparisons.

### 2.5 Weighted logistic regression analysis

Weighted binary logistic regression was utilized to investigate explore the possible association between levels of TPA and the occurrence of MASLD-HCC. In the regression model, TPA levels were incorporated as both continuous and categorical variables. This methodological framework enabled the estimation of odds ratios (ORs) along with their respective 95% confidence intervals (95% CIs). The TPA concentrations were first treated as a continuous variable. Subsequently, these concentrations were categorized into quartiles according to the interquartile range, organized from the lowest to the highest levels. These quartiles were designated as the first quartile (Q1), second quartile (Q2), third quartile (Q3), and fourth quartile (Q4), and were also assessed as categorical variables. In cases where TPA levels were analyzed as categorical variables, the lowest quartile (Q1) served as the reference group. The study delineated three distinct statistical analysis groups: model 1 constituted the unadjusted model, whereas model 2 incorporated adjustments for demographic factors including gender, age, race, education, and poverty-income ratio (PIR). Building on the adjustments made in model 2, model 3 additionally accounted for factors such as diabetes, lipid profiles, cholesterol levels, low-density lipoprotein, high-density lipoprotein, albumin, creatinine, blood urea nitrogen, hemoglobin, platelet count, and glycated hemoglobin. All regression analyses were conducted with the inclusion of survey weights, and non-normally distributed continuous covariates underwent transformation utilizing weighted quartiles. Furthermore, the study evaluated potential interactions among factors associated with TPA levels through the application of the variance inflation factor (VIF), where a VIF value of less than 10 indicated no interaction between the examined factor and other variables. The *P* values were adjusted to account for the false discovery rate (FDR).

### 2.6 Clinical subgroup analysis

Forest plots are primarily used for visualizing the comparison of effect sizes and CIs across multiple study results. They are commonly employed to summarize and compare outcomes from different studies, providing a more intuitive representation of the effect size (such as RR, OR, HR, or WMD) and their corresponding 95% CIs. This visualization aids in better understanding the consistency and discrepancies between different studies.

### 2.7 Restricted cubic spline

Restricted cubic spline is a concept in statistics, particularly frequently used in regression analysis and curve fitting. It is a method for fitting and modeling continuous variables by dividing the data range into several intervals and using a cubic polynomial for fitting within each interval to create a smooth curve. These polynomials are smoothly connected across adjacent intervals, often with additional smoothness constraints to avoid sharp fluctuations in the curve. In statistical modeling, RCS is commonly used to model the relationship between continuous variables and the dependent variable. In regression analysis, it allows for capturing nonlinear relationships while maintaining smoothness and avoiding overfitting. In this study, the RCS analysis was performed using the R “rms” package, with three knots automatically selected as the optimal number based on statistical algorithms.

### 2.8 Multilevel logistic regression model

In the realm of statistics, the multilevel logistic regression model serves as an advanced classification technique that extends traditional logistic regression to accommodate multicategory scenarios. This model is particularly designed to forecast the probabilities associated with various potential outcomes of a dependent variable that has a categorical distribution. Within the framework of a multilevel logistic regression model, the dependent variable’s predictions are made utilizing a series of independent variables, also known as features or observed variables. This model operates by computing the likelihood of achieving a specific outcome in the dependent variable through a linear combination of the independent variables along with their respective parameters, all framed within a probabilistic model. The parameters linked to the independent variables are derived from the training dataset, and they are commonly referred to as regression coefficients. Importantly, the multilevel logistic regression model empowers researchers to account for the inherent clustered structure of the data, explore the origins of variation both within and between clusters, identify which variables account for individual differences, and ascertain which factors influence variations at the cluster level.

### 2.9 Statistical analysis

All data processing and analytical procedures were performed utilizing R software (version 4.4.0). For the analysis of baseline characteristics, medians and interquartile ranges were utilized to characterize continuous variables that exhibited non-normal distributions. Classification variables were reported as sample counts and weighted percentages. To check for changes in variable characteristics between groups of TPA (quartiles), we used Wilcoxon rank sum tests to test continuous variables and Rao-Scott Chi-square tests to test weighted percentages of categorical variables, providing a comprehensive description of the entire population. The “mediation” package was used for mediation analysis to assess the mediating effects of lipid indicators (including cholesterol, low density lipoprotein, high density lipoprotein, and triglycerides) on the association between TPA and MASLD-HCC. The existence of mediation effects was characterized by the presence of a notable indirect effect, a significant overall effect, and a positive ratio of the mediation effect. All statistical evaluations were conducted as two-tailed tests, with a *P*-value of less than 0.05 deemed to indicate statistical significance. For all hypothesis tests (including primary, subgroup, and mediation analyses), Benjamini–Hochberg method was applied to adjust *P* values for FDR.

## 3 Results

### 3.1 Baseline characteristics of the participants

In this study, a final cohort of 548 patients was incorporated to evaluate the baseline characteristics, including factors such as gender, age, and race, in connection with the occurrence of HCC. The baseline characteristics of participants were presented in Table 1. Before PSM, education and smoking showed differences between the HCC and non-HCC groups (*P*-value = 0.038, *P*-value = 0.039), whereas no differences were observed after PSM (*P*-value = 0.1, *P*-value = 0.87). Some other baseline data also showed differences before and after PSM in both the HCC and non-HCC groups, such as race (*P*-value < 0.001 before PSM, *P*-value = 0.026 after PSM), BMI (*P*-value = 0.003 before PSM, *P*-value = 0.002 after PSM), diabetes (*P*-value = 0.013 before PSM, *P*-value = 0.027 after PSM), total cholesterol (*P*-value < 0.001 before PSM, *P*-value < 0.001 after PSM), triglycerides (*P*-value = 0.015 before PSM, *P*-value = 0.039 after PSM), and low-density lipoprotein (*P*-value = 0.001 before PSM, *P*-value < 0.001 after PSM). These indicators, which showed differences before and after PSM in both the HCC and non-HCC groups, could be considered for mediation effect analysis. Some baseline indicators, however, showed no differences before and after PSM in both the HCC and non-HCC groups, such as gender, age, education, and PIR. This indicates that these indicators were relatively balanced between the HCC and non-HCC groups, and their confounding factors were excluded when comparing the two groups.

**TABLE 1:** Baseline characteristics of participants.

Variable	Before PSM				After PSM		
	Overall	Liver cancer	Normal	*P*-value^2^	Liver cancer	Normal	*P*-value^2^
	*N* = 548^1^	*N* = 239^1^	*N* = 309^1^		*N* = 239^1^	*N* = 239^1^	
Gender				0.36			0.17
Male	279 (51%)	127 (53%)	152 (49%)		127 (53%)	112 (47%)	
Female	269 (49%)	112 (47%)	157 (51%)		112 (47%)	127 (53%)	
Age (years)	53 (16)	54 (16)	52 (16)	0.17	54 (16)	52 (16)	0.1
Race				< 0.001			0.026
Mexican American	100 (18%)	61 (26%)	39 (13%)		61 (26%)	39 (16%)	
Other Hispanic	40 (7.3%)	11 (4.6%)	29 (9.4%)		11 (4.6%)	4 (1.7%)	
Non-Hispanic White	263 (48%)	107 (45%)	156 (50%)		107 (45%)	127 (53%)	
Non-Hispanic Black	125 (23%)	54 (23%)	71 (23%)		54 (23%)	59 (25%)	
Others	20 (3.6%)	6 (2.5%)	14 (4.5%)		6 (2.5%)	10 (4.2%)	
Education				0.038			0.1
Less than 9th grade	58 (11%)	36 (15%)	22 (7.1%)		36 (15%)	17 (7.1%)	
9th–11th grade	80 (15%)	37 (15%)	43 (14%)		37 (15%)	40 (17%)	
High school	142 (26%)	60 (25%)	82 (27%)		60 (25%)	66 (28%)	
AA degree	176 (32%)	70 (29%)	106 (34%)		70 (29%)	77 (32%)	
College graduate	92 (17%)	36 (15%)	56 (18%)		36 (15%)	39 (16%)	
PIR				0.17			0.14
High	393 (79%)	172 (82%)	221 (76%)		172 (82%)	168 (76%)	
Low	107 (21%)	39 (18%)	68 (24%)		39 (18%)	54 (24%)	
BMI	34.8 (6.0)	34.0 (5.8)	35.4 (6.2)	0.003	34.0 (5.8)	35.7 (6.4)	0.002
Smoke				0.039			0.87
Regularly	117 (21%)	61 (26%)	56 (18%)		61 (26%)	56 (23%)	
Sometimes	33 (6%)	9 (3.8%)	24 (7.8%)		9 (3.8%)	9 (3.8%)	
Never	398 (73%)	169 (71%)	229 (74%)		169 (71%)	174 (73%)	
Hypertension				0.17			0.2
Hypertension	266 (49%)	108 (45%)	158 (51%)		108 (45%)	122 (51%)	
Health	282 (51%)	131 (55%)	151 (49%)		131 (55%)	117 (49%)	
Diabetes				0.013			0.027
Diabetes	73 (13%)	22 (9.2%)	51 (17%)		22 (9.2%)	38 (16%)	
Health	475 (87%)	217 (91%)	258 (83%)		217 (91%)	201 (84%)	
Heart failure				0.52			0.5
Heart failure	24 (4.4%)	12 (5.0%)	12 (3.9%)		12 (5.0%)	9 (3.8%)	
Health	524 (96%)	227 (95%)	297 (96%)		227 (95%)	230 (96%)	
CHD				0.93			> 0.99
CHD	27 (4.9%)	12 (5.0%)	15 (4.9%)		12 (5.0%)	12 (5.0%)	
Health	521 (95%)	227 (95%)	294 (95%)		227 (95%)	227 (95%)	
Fasting insulin (μU/ml)	20 (15)	20 (19)	20 (11)	0.13	20 (19)	20 (11)	0.071
TC (mmol/L)	5.29 (1.07)	5.47 (1.05)	5.15 (1.07)	< 0.001	5.47 (1.05)	5.15 (1.06)	< 0.001
TG (mmol/L)	1.97 (1.41)	2.10 (1.50)	1.86 (1.33)	0.015	2.10 (1.50)	1.89 (1.40)	0.039
LDL-C (mmol/L)	3.25 (0.98)	3.40 (0.92)	3.13 (1.02)	0.001	3.40 (0.92)	3.12 (1.00)	< 0.001
UACR (mg/g)	0.68 (7.50)	1.17 (11.25)	0.29 (1.29)	0.05	1.17 (11.25)	0.26 (1.02)	0.14
ALB (g/L)	42.2 (3.3)	43.4 (3.2)	41.2 (3.1)	< 0.001	43.4 (3.2)	41.1 (3.2)	< 0.001
Cr (μmol/L)	75 (42)	68 (48)	81 (35)	< 0.001	68(48)	81 (38)	< 0.001
BUN (mg/dl)	14.4 (6.0)	14.9 (5.0)	14.1 (6.6)	0.003	14.9 (5.0)	13.7 (6.1)	< 0.001
WBC (%)	7.12 (1.94)	7.10 (1.93)	7.13 (1.95)	0.9	7.10 (1.93)	7.18 (1.95)	0.87
Neutrophils (%)	4.26 (1.59)	4.24 (1.54)	4.26 (1.63)	0.72	4.24 (1.54)	4.29 (1.59)	0.97
Lymphocytes (%)	2.08 (0.68)	2.07 (0.66)	2.08 (0.70)	0.74	2.07 (0.66)	2.09 (0.70)	0.58
HB (g/dl)	14.36 (1.46)	14.58 (1.42)	14.19 (1.48)	0.004	14.58 (1.42)	14.21 (1.49)	0.015
PLT (%)	250 (63)	259 (61)	243 (64)	0.002	259 (61)	242 (66)	< 0.001
HBA1c (%)	5.89 (1.07)	5.89 (1.32)	5.88 (0.83)	0.002	5.89 (1.32)	5.87 (0.83)	0.006
TPA (μmol/L)	6.8 (3.8)	8.6 (4.0)	4.9 (2.4)	< 0.001	8.6 (4.0)	5.0 (2.6)	< 0.001

PIR < 1.2: low; PIR ≥ 1.2: high. PIR, poverty-to-income ratio; BMI, body mass index; CHD, coronary heart disease; TC, total cholesterol; TG, triglyceride; LDL-C, low-density lipoprotein cholesterol; UACR, urinary albumin/creatinine ratio; ALB, albumin; Cr, creatinine; BUN, blood urea nitrogen; WBC, white blood cell count; HB, hemoglobin; PLT, platelet count; HBA1c, hemoglobin A1c; TPA, trans-palmitoleic acid; PSM, propensity score matching.

^1^Mean (SD) or frequency (%).

^2^Pearson’s Chi-squared test; Wilcoxon rank sum test.

### 3.2 Association of MASLD-HCC with TPA

**FIGURE 2:**
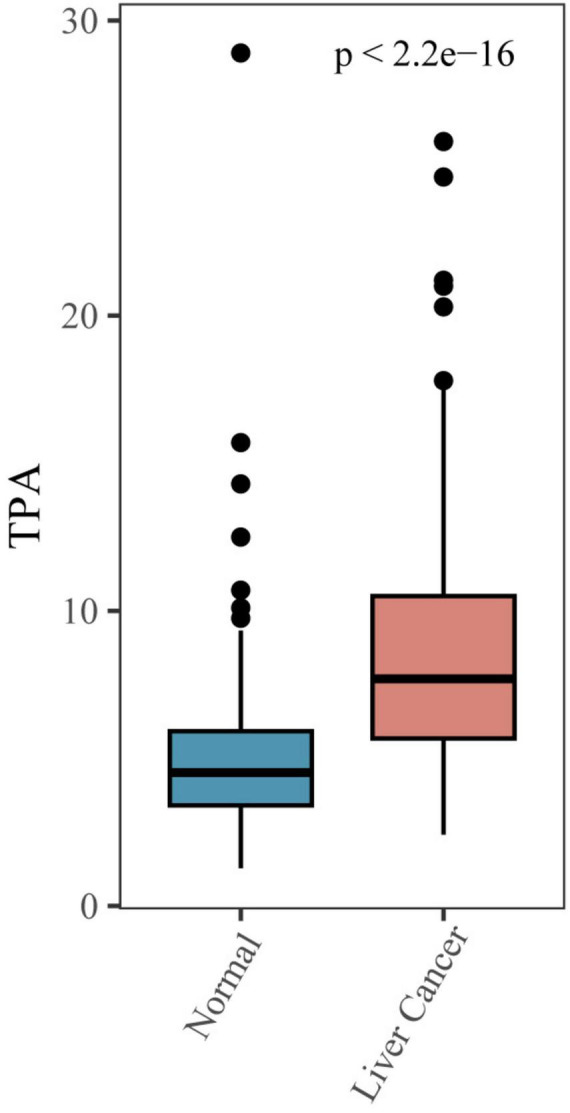
Bar chart of TPA serum level between MASLD-HCC and non-cancerous group.

We utilized box plots to illustrate the relationship between MASLD-HCC and TPA, revealing a significant difference in TPA levels between the cancer and non-cancer groups (Figure 2). To further analyze this difference, we utilized linear regression analysis and multilevel logistic regression model to demonstrate the relationship (Table 2). Model 1, without covariates, revealed a positive correlation between TPA levels and MASLD-HCC (OR = 1.524, 95% CI = 1.397–1.677, *P*-value < 0.001); when compared to Q1, Q2 had an OR of 3.671 (95% CI = 1.997–6.987, *P*-value < 0.001), Q3 had an OR of 7.519 (95% CI = 4.132–14.258, *P*-value < 0.001), and Q4 had an OR of 42.905 (95% CI = 20.946–94.294, *P*-value < 0.001), indicating that with each increase in TPA, the probability of developing MASLD-HCC increased. Model 2, with gender, age, race, education, and PIR as covariates, and model 3, further adding diabetes, blood lipids, cholesterol, low-density lipoprotein, high-density lipoprotein, albumin, creatinine, blood urea nitrogen, hemoglobin, platelet count, and glycated hemoglobin as covariates, both showed a similar positive correlation between TPA and MASLD-HCC (model 2 OR = 1.654, 95% CI = 1.487–1.862, *P*-value < 0.001; model 3 OR = 2.276, 95% CI = 1.921–2.767, *P*-value < 0.001). When grouped by quartile intervals, both model 2 and model 3 revealed that, compared to Q1, the OR values for Q2 (model 2 OR = 6.170, 95% CI = 2.983–13.500, *P*-value < 0.001; model 3 OR = 9.500, 95% CI = 4.069–23.801, *P*-value < 0.001), Q3 (model 2 OR = 15.239, 95% CI = 7.183–34.719, *P*-value < 0.001; model 3 OR = 26.202, 95% CI = 10.721–70.020, *P*-value < 0.001), and Q4 (model 2 OR = 110.166, 95% CI = 44.800–296.784, *P*-value < 0.001; model 3 OR = 753.702, 95% CI = 196.665–3,490.227, *P*-value < 0.001) increased progressively, indicating that with the addition of covariates, the probability of developing MASLD-HCC also increased with increasing TPA.

**TABLE 2:** Relationship between TPA and promotion of MASLD-HCC development.

	Model 1			Model 2			Model 3		
Characteristic	OR	95% CI	*P*-value	OR	95% CI	*P*-value	OR	95% CI	*P*-value
TPA continuous	1.524	(1.397, 1.677)	*P* < 0.001	1.654	(1.487, 1.862)	*P* < 0.001	2.276	(1.921, 2.767)	*P* < 0.001
**TPA quantile**									
Q1 (low)	Ref	Ref		Ref	Ref		Ref	Ref	
Q2	3.671	(1.997, 6.987)	*P* < 0.001	6.17	(2.983, 13.5)	*P* < 0.001	9.5	(4.069, 23.801)	*P* < 0.001
Q3	7.519	(4.132, 14.258)	*P* < 0.001	15.239	(7.183, 34.719)	*P* < 0.001	26.202	(10.721, 70.02)	*P* < 0.001
Q4 (high)	42.905	(20.946, 94.294)	*P* < 0.001	110.166	(44.8, 296.784)	*P* < 0.001	753.702	(196.665, 3,490.227)	*P* < 0.001
*P*-value for trend			*P* < 0.001			*P* < 0.001			*P* < 0.001

Q1 (low) < 4.1, 4.1 ≤ Q2 < 5.83, 5.83 ≤ Q3 < 8.29, and Q4 ≥ 8.29. The model 1 was the crude model. The model 2 was adjusted by gender, age, race, education, and PIR. The model 3 was adjusted by gender, age, race, education, PIR, diabetes, TC, TG, LDL_C, ALB, SCR, BUN, HB, PLT, and HBA1C. TPA, trans-palmitoleic acid.

### 3.3 Restricted cubic spline curves for MASLD-HCC and its subgroups

**FIGURE 3:**
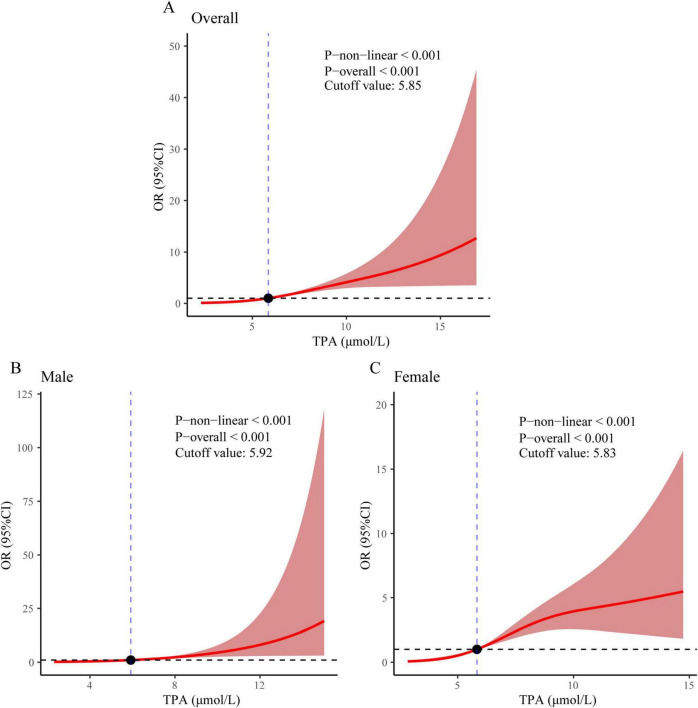
Restricted cubic spline regression analysis revealed an association between MASLD-HCC and TPA levels in **(A)** the overall population, **(B)** males, and **(C)** females.

Restricted cubic spline curves were utilized to explore the association between TPA levels and the incidence of MASLD-HCC, while accounting for all pertinent covariates. A statistically significant nonlinear relationship was observed between TPA and MASLD-HCC (*P* non-linear < 0.001, cutoff value = 5.85 μmol/L, Figure 3A). Furthermore, a significant nonlinear relationship was found between TPA and the incidence of MASLD-HCC in the male subgroup (*P* non-linear < 0.001, cutoff value = 5.92 μmol/L, Figure 3B), as well as in the female subgroup (*P* non-linear < 0.001, cutoff value = 5.83 μmol/L, Figure 3C).

### 3.4 Relationship of TPA with baseline characteristics in various subgroups

**FIGURE 4:**
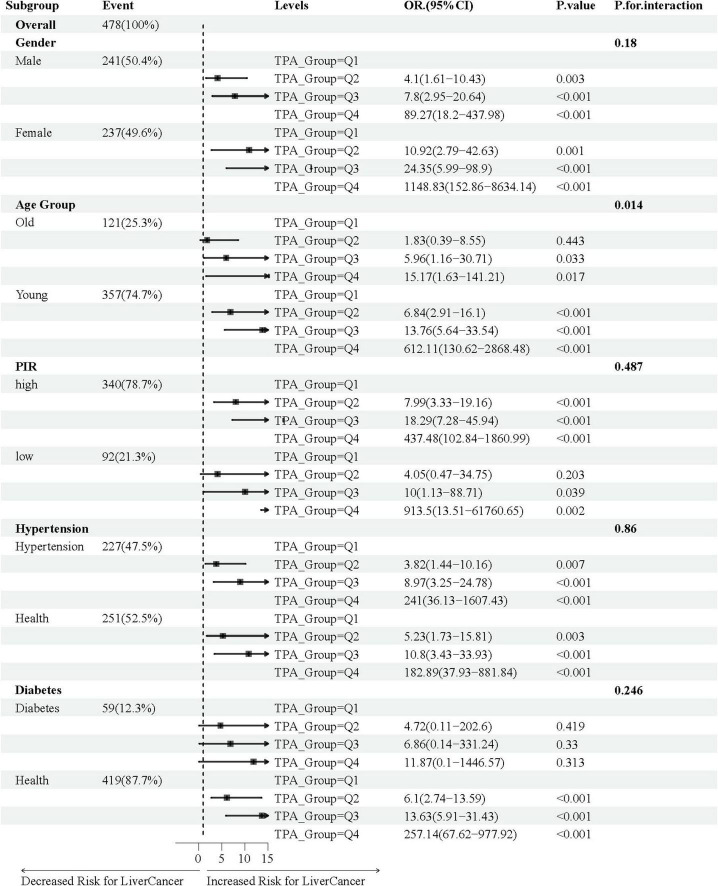
Association of TPA with MASLD-HCC in various subgroups of baseline characteristics. In the age subgroup, “Young” refers to age ≤ 65 years, and “Old” refers to age > 65 years; in the poverty-income ratio (PIR) subgroup, “high” is defined as PIR ≥ 1.2, and “low” is defined as PIR < 1.2.

Figure 4 illustrates the association between TPA and MASLD-HCC, with the relationship analyzed across various subgroups stratified by gender, age, PIR, hypertension, and diabetes using fully adjusted multivariate logistic regression. We found that in both male and female gender subgroups, the incidence of HCC increased with increasing levels of TPA (male group: Q2 vs. Q1, OR = 4.10, 95% CI = 1.61–10.43, *P*-value = 0.003; Q3 vs. Q1, OR = 7.80, 95% CI = 2.95–20.64, *P*-value < 0.001; Q4 vs. Q1, OR = 89.27, 95% CI = 18.20–437.98, *P*-value < 0.001; female group: Q2 vs. Q1, OR = 10.92, 95% CI = 2.79–42.63, *P*-value = 0.001; Q3 vs. Q1, OR = 24.35, 95% CI = 5.99–98.90, *P*-value < 0.001; Q4 vs. Q1, OR = 1,148.83, 95% CI = 152.86–8,634.14, *P*-value < 0.001). The age young subgroup appeared to be more sensitive to TPA, with increasing OR values for Q2, Q3, and Q4 compared to Q1 (Q2 vs. Q1, OR = 6.84, 95% CI = 2.91–16.10, *P*-value < 0.001; Q3 vs. Q1, OR = 13.76, 95% CI = 5.64–33.54, *P*-value < 0.001; Q4 vs. Q1, OR = 612.11, 95% CI = 130.62–2,868.48, *P*-value < 0.001), while the tendency was observed in the age old subgroup only when comparing Q3 and Q4 to Q1 (Q3 vs. Q1, OR = 5.96, 95% CI = 1.16–30.71, *P*-value = 0.033; Q4 vs. Q1, OR = 15.17, 95% CI = 1.63–141.21, *P*-value = 0.017). A similar trend was observed in the PIR subgroup, with significant differences in Q2, Q3, and Q4 compared to Q1 in the high PIR group (Q2 vs. Q1, OR = 7.99, 95% CI = 3.33–19.16, *P*-value < 0.001; Q3 vs. Q1, OR = 18.29, 95% CI = 7.28–45.94, *P*-value < 0.001; Q4 vs. Q1, OR = 437.48, 95% CI = 102.84–1,860.99, *P*-value < 0.001), and in Q3 and Q4 compared to Q1 in the low PIR group (Q3 vs. Q1, OR = 10.00, 95% CI = 1.13–88.71, *P*-value = 0.039; Q4 vs. Q1, OR = 913.50, 95% CI = 13.51–61,760.65, *P*-value = 0.002). In the hypertension subgroup, an increase in the incidence of HCC was observed with increasing levels of TPA, regardless of whether the individual had hypertension. In the diabetes subgroup, patients without diabetes appeared to be more sensitive to TPA levels (Q2 vs. Q1, OR = 6.10, 95% CI = 2.74–13.59, *P*-value < 0.001; Q3 vs. Q1, OR = 13.63, 95% CI = 5.91–31.43, *P*-value < 0.001; Q4 vs. Q1, OR = 257.14, 95% CI = 67.62–977.92, *P*-value < 0.001).

### 3.5 Association of metabolic indicators with TPA and MASLD-HCC

Table 3 presents the associations of TPA with metabolic indicators following multivariate logistic regression. Upon controlling for all potential confounding variables, a positive correlation was observed between TPA and cholesterol levels (β = 0.644, 95% CI = 0.474–0.819, *P*-value < 0.001), triglycerides (β = 1.236, 95% CI = 0.994–1.493, *P*-value < 0.001), low-density lipoprotein (β = 0.557, 95% CI = 0.372–0.749, *P*-value < 0.001), albumin (β = 0.138, 95% CI = 0.08–0.198, *P*-value < 0.001), hemoglobin (β = 0.148, 95% CI = 0.009–0.291, *P*-value = 0.036), and platelet count (β = 0.004, 95% CI = 0–0.007, *P*-value = 0.007). It was negatively correlated with BMI (β = −0.037, 95% CI = −0.068–0.006, *P*-value = 0.021).

**TABLE 3:** Associations between TPA and metabolic related indicators.

	β value	95% CI	*P*-value
**BMI**			
Model 1	−0.038	(−0.065, −0.011)	0.006
Model 2	−0.045	(−0.075, −0.016)	0.002
Model 3	−0.037	(−0.068, −0.006)	0.021
**TC**			
Model 1	0.642	(0.48, 0.808)	*P* < 0.001
Model 2	0.644	(0.482, 0.811)	*P* < 0.001
Model 3	0.644	(0.474, 0.819)	*P* < 0.001
**TG**			
Model 1	1.205	(0.98, 1.443)	*P* < 0.001
Model 2	1.209	(0.984, 1.447)	*P* < 0.001
Model 3	1.236	(0.994, 1.493)	*P* < 0.001
**LDL_C**			
Model 1	0.543	(0.367, 0.724)	*P* < 0.001
Model 2	0.544	(0.368, 0.725)	*P* < 0.001
Model 3	0.557	(0.372, 0.749)	*P* < 0.001
**ALB**			
Model 1	0.123	(0.072, 0.176)	*P* < 0.001
Model 2	0.15	(0.094, 0.207)	*P* < 0.001
Model 3	0.138	(0.08, 0.198)	*P* < 0.001
**Cr**			
Model 1	−0.005	(−0.012, 0.001)	0.039
Model 2	−0.006	(−0.014, 0.001)	0.032
Model 3	−0.001	(−0.009, 0.006)	0.644
**BUN**			
Model 1	0.004	(−0.026, 0.034)	0.805
Model 2	0.003	(−0.03, 0.038)	0.846
Model 3	0.013	(−0.022, 0.049)	0.469
**HB**			
Model 1	0.167	(0.06, 0.277)	0.002
Model 2	0.257	(0.127, 0.39)	*P* < 0.001
Model 3	0.148	(0.009, 0.291)	0.036
**PLT**			
Model 1	0.003	(0, 0.006)	0.011
Model 2	0.003	(0, 0.007)	0.01
Model 3	0.004	(0, 0.007)	0.007
**HBA1C**			
Model 1	0.038	(−0.122, 0.194)	0.635
Model 2	0.037	(−0.128, 0.198)	0.651
Model 3	0.166	(−0.037, 0.369)	0.109

The model 1 was the crude model. The model 2 was adjusted by gender, age, race, education, and PIR. The model 3 was adjusted by gender, age, race, education, PIR, diabetes, TC, TG, LDL_C, ALB, SCR, BUN, HB, PLT, and HBA1C. BMI, body mass index; TC, total cholesterol; TG, triglyceride; LDL-C, low-density lipoprotein cholesterol; ALB, albumin; Cr, creatinine; BUN, blood urea nitrogen; HB, hemoglobin; PLT, platelet count; HBA1c, hemoglobin A1c.

### 3.6 Analysis of the mediating role of metabolic indicators in the association with TPA

**FIGURE 5:**
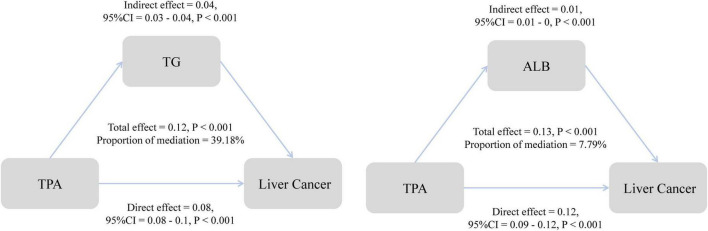
Mediating effects of triglycerides and albumin.

Building upon the results of the analysis of metabolic indicators, we conducted further mediation analysis to investigate the role of these indicators in the association between TPA and MASLD-HCC. The study revealed that in the mediation by triglycerides, the direct effect of TPA on HCC was 0.08, the indirect effect was 0.04, and the total effect was 0.12. Triglycerides mediated 39.18% of the association between TPA and the incidence of HCC (Figure 5). In the case of albumin, the direct effect was 0.12, the indirect effect was 0.01, and the total effect was 0.13, with a mediating proportion of 7.79%. Additionally, we evaluated the mediating roles of various metabolic parameters, including cholesterol and low-density lipoprotein, in the association (Supplementary Table 1).

## 4 Discussion

The present study provides compelling evidence that TPA serves as an independent risk factor for HCC in patients with MASLD, with a striking nonlinear dose-response relationship. Our findings significantly advance the current understanding of MASLD-HCC pathogenesis by identifying TPA as a novel biomarker and elucidating its metabolic mediation pathways. Unlike previous studies that primarily focused on traditional risk factors such as diabetes (35, 36), our research highlights the unique role of TPA in driving hepatocarcinogenesis, particularly through its association with triglycerides (39.18% mediation effect). Notably, the exponential risk escalation in high-TPA quartiles (Q4 OR = 753.7) and pronounced gender disparity (female Q4 OR = 1,148.83) represent previously unrecognized dimensions of MASLD-HCC susceptibility, challenging the conventional paradigm of metabolic liver disease progression.

Clinically, these findings could revolutionize risk stratification and early intervention strategies for MASLD patients. The robust association between TPA and HCC, coupled with its measurable metabolic correlates (e.g., triglycerides β = 1.236), suggests that routine TPA monitoring could identify high-risk cohorts years before malignant transformation. This is particularly relevant given the limitations of current surveillance tools like ultrasound elastography, which often detect late-stage fibrosis. Notably, our subgroup analyses revealed that females and younger individuals exhibit greater susceptibility to TPA-mediated hepatocarcinogenesis—possibly associated with estrogen-modulated inflammatory pathways and age-related declines in hepatic regenerative capacity. Our RCS analysis further provides actionable thresholds for risk mitigation—the nonlinear inflection points could guide dietary modifications (e.g., reducing dairy/meat-derived TPA) or pharmacologic interventions targeting triglyceride metabolism. TPA also offers several practical advantages, such as low assay cost, rapid turnaround time, and predictable gender- and age-specific risk patterns. For policymakers, these data underscore the need to reevaluate nutritional guidelines in MASLD management, as TPA’s dual role as both a biomarker and modifiable risk factor bridges diagnostic and therapeutic gaps in this burgeoning epidemic.

Current investigations into the relationship between dietary trans fatty acids and the risk of developing cancer are significantly limited, primarily concentrating on trans-11-octadecenoic acid (VA) and trans-9,11,15-octadecatrienoic acid [conjugated linoleic acid (CLA)]. A cohort study conducted in the Netherlands indicated a potential link between the consumption of VA and an elevated risk of breast cancer (37). In addition, another epidemiological investigation established a direct relationship between VA levels in serum or red blood cells and the incidence of both breast and prostate cancers (38, 39). In terms of CLA’s connection to cancer risk, four case-control studies have been performed. One of the investigations revealed a negative correlation between the consumption of CLA and the risk of developing colorectal cancer (40). Another research effort demonstrated that postmenopausal women with breast cancer exhibited significantly lower dietary CLA intake and serum CLA levels than their counterparts without breast cancer (41). It is especially noteworthy that women identified in the uppermost quartile of CLA intake demonstrated a 29% lower risk of developing colorectal cancer in comparison to those in the lowest quartile. Conversely, two other case-control studies did not establish a noteworthy relationship between CLA intake, whether through diet (42) or CLA concentrations in adipose tissue (43), and the risk of breast cancer. Moreover, a prospective cohort analysis indicated a weak association between CLA intake and the incidence of breast cancer, a conclusion derived from a comparison of the highest and lowest quintiles of CLA consumption (37).

Several limitations warrant consideration. First, despite rigorous PSM, residual confounding from unmeasured metabolic variables (e.g., adipose tissue distribution) may persist, though the consistency of effects across multivariable models strengthens causal inference. Second, the observational design precludes mechanistic validation; future studies should employ experimental models to delineate whether TPA directly induces oncogenic mutations or acts via microenvironmental remodeling. Third, while NHANES provides nationally representative data, batch effects from pooled survey cycles may introduce measurement variability. Forth, although FLI enabled MASLD classification in NHANES, its limitations in HCC risk stratification must be noted. Future studies should incorporate imaging and biopsy examinations in MASLD diagnosis. Finally, the modest sample size (*n* = 548) limited statistical power for subgroup analyses. Addressing these through multicenter cohorts with serial TPA measurements and -omics integration will be critical for translating these findings into precision prevention frameworks. Nevertheless, this study establishes TPA as a pivotal player in MASLD-HCC pathogenesis, opening new avenues for risk prediction and targeted metabolic therapy.

## 5 Conclusion

This study establishes TPA as a robust, nonlinear risk factor for MASLD-HCC, with effect sizes exceeding conventional metabolic indicators. The pronounced gender/age disparities and the 39.18% mediation by triglycerides underscore TPA’s dual role as a biomarker and potential therapeutic target. Despite methodological constraints, these findings advocate for clinical trials testing TPA-lowering strategies in high-risk subgroups, while highlighting the need for mechanistic studies to decode its oncogenic pathways in hepatic metabolic reprogramming.

## Data Availability

The datasets presented in this study can be found in online repositories. The names of the repository/repositories and accession number(s) can be found below: https://wwwn.cdc.gov/nchs/nhanes/.
